# Electron-Beam Irradiation of the PLLA/CMS/β-TCP Composite Nanofibers Obtained by Electrospinning

**DOI:** 10.3390/polym12071593

**Published:** 2020-07-17

**Authors:** Mohd Reusmaazran Yusof, Roslinda Shamsudin, Sarani Zakaria, Muhammad Azmi Abdul Hamid, Fatma Yalcinkaya, Yusof Abdullah, Norzita Yacob

**Affiliations:** 1 Faculty of Sciences and Technology, National University of Malaysia, Bandar Baru Bangi, 43600 Selangor, Malaysia; linda@ukm.edu.my (R.S.); szakaria@ukm.edu.my (S.Z.); norzita@nuclearmalaysia.gov.my (N.Y.); 2 Institute for Nanomaterials, Advanced Technology and Innovation, Technical University of Liberec, Studentska 1402/2, 46117 Liberec, Czech Republic; 3 Material Technology Group, Malaysian Nuclear Agency, Bangi, Kajang, 43300 Selangor, Malaysia; yusofabd@nm.gov.my

**Keywords:** PLLA, carboxy-methyl starch, β-tricalcium phosphate, electrospun, biodegradation

## Abstract

Nanofibrous materials produced by electrospinning processes have potential advantages in tissue engineering because of their biocompatibility, biodegradability, biomimetic architecture, and excellent mechanical properties. The aim of the current work is to study the influence of the electron beam on the poly L-lactide acid/ carboxy-methyl starch/β-tricalcium phosphate (PLLA/CMS/β-TCP) composite nanofibers for potential applications as bone-tissue scaffolds. The composite nanofibers were prepared by electrospinning in the combination of 5% *v*/*v* carboxy-methyl starch (CMS) and 0.25 wt% of β-TCP with the PLLA as a matrix component. The composites nanofibers were exposed under 5, 30, and 100 kGy of irradiation dose. The electron-beam irradiation showed no morphological damage to the fibers, and slight reduction in the water-contact angle and mechanical strength at the higher-irradiation doses. The chain scission was found to be a dominant effect; the higher doses of electron-beam irradiation thus increased the in vitro degradation rate of the composite nanofibers. The chemical interaction due to irradiation was indicated by the Fourier transform infrared (FTIR) spectrum and thermal behavior was investigated by a differential scanning calorimeter (DSC). The results showed that the electron-beam-induced poly L-lactide acid/carboxy-methyl starch/β-tricalcium phosphate (PLLA/CMS/β-TCP) composite nanofibers may have great potential for bone-tissue engineering.

## 1. Introduction

Nanofibers have acquired huge interest in the past few decades due their great properties that can be used in a wide range of applications. Nanofibers can provide great surface area over volume, high connected pores, and good mechanical properties [1,2,3,4]. Due to their unique properties, nanofibers have become potential candidates that can be used in various fields such as filtration, electronics, textile, tissue engineering, and drug-delivery systems [5,6,7,8,9,10,11,12,13]. The research interests into the application of nanofiber mats is increasing every year. Nanofibers can be obtained by several techniques, including drawing, self-assembly, synthesis techniques, and electrospinning [14]. Among them, electrospinning has become the most popular technique because of its economic properties, easy-to-control parameters, and versatility for producing nanofibers [15,16,17]. Either polymeric melt or the polymeric solution can be used in the electrospinning technique [18,19]. In early studies, a single polymer was used to produce nanofibers [20]. Due to the high demand for the technology, composite polymers have gained more attention over the past decade, either as synthetic–synthetic or synthetic–natural polymers. Additional nanoparticles in nanofibers enhance the properties for multiple applications such as in the electronic industries [21,22].

In tissue engineering of nanofibers, cell attachment and proliferation is the most important aspect that needs to be considered when developing new scaffold material [23]. Besides biocompatibility, the transport of nutrients and waste through the body are among the factors that need to be studied. Therefore, the used nanofiber scaffold must not remain in the body for long periods, and materials must degrade by growing new tissue. The incorporation of natural polymers into the synthetic ones was proved to modify the in vitro degradation of nanofibers. Zulkifli et al. [24] reported that in vitro degradation was modified by the incorporation of collagen into the HEC/PVA in both Dulbecco’s Modified Eagle’s medium (DMEM) and phosphate buffer solutions (PBS). Mixing PLLA with aniline pentamer-graft-gelatine was found to increase the mass loss compared to neat PLLA nanofibers [13]. Besides providing hydrophilic properties to natural polymers, incorporating natural polymers with synthetic ones produces different morphologies that help the nanofibers to degrade. Shi et al. [25] observed the increase in degradation rate after cellulose nanocrystal (CNC) was incorporated with PLA. The mass loss increased from 14.7% to 38.9% of neat PLA and 10% CNC, respectively.

The effect of ionizing radiation on PLLA has been reported by several authors, either by gamma or electron beam [26,27,28]. Commonly, irradiation agents, such as triallyl isocyanurate, were used to improve strength by crosslinking the polymer chain. However, crosslinking reduces the in vitro degradation rate of the PLLA. According to Lee et al. [29], PLGA nanofibers underwent mass loss of about 80% after 6 weeks of incubation in PBS, if irradiation was more than 150 kGy with an electron beam. Mechanical strength was reduced from 10 ± 0.7 MPa to 5.8 ± 0.8 MPa after PLGA nanofibers were irradiated under 150 kGy due to the polymer chain degradation. Similar results have been reported by Loo et al. [30] on PLGA and PLLA. A chain scission was dominant compared to crosslinking, even though there was a recombination of the trapped shorter chain, which would occur in the crystalline phase. The effect of gamma irradiation on chitosan/PVA nanofibers was observed by Jeun et al. [31]. According to Jeun, at higher doses of irradiation, degradation of the intermolecular hydrogen bond between chitosan and PVA was demonstrated by a sharp and large peak in the DSC thermogram. The degradation of chitosan/PVA was also confirmed by a reduction in mechanical strength. A decrease in nanofiber diameter of PLLA/PDLA blend from 522 ± 105 to 432 ± 103 nm was observed by Zhang et al. [32] under electron-beam irradiation up to 100 kGY. A DSC thermogram also shifted to a lower melting temperature, indicating a severe degradation by chain scission. However, Zhang observed an increase in hydrophilic surface by decreasing water-contact angle. In nanofibers, the crystalline region is retarded during electrospinning due to fast cooling. The polymer chain does not rearrange itself in a crystalline structure [33]. The degradation of the polymer chain can be observed in the reduction in mechanical strength and molecular weight. 

In this work, we used an electron beam to study the properties of composite natural–synthetic nanofibers. The incorporation was a natural polymer of carboxy-methyl starch (CMS) derived from a sago starch with PLLA. The CMS provided the carbohydrate source, as present in the extra-cellular matrixes (ECM). CMS consists of amylose and amylopectin in its molecular structure with a 1–4 glycosidic bond that can enhance cell and material interaction. β-TCP particles were used to initiate the potential of this composite nanofiber to be used in bone-tissue engineering. The presence of β-TCP particles in the nanofibers would enhance the initiating mechanism of the cell interaction. β-TCP particles have a rough surface morphology that encourages attachment to bone cells. This study is an extension of our previous work in composite nanofibers. The previous study was carried out to determine the ratio between PLLA/CMS and PLLA/CMS/β-TCP to investigate the effect of the mix ratio of all components [4,34]. The physical, chemical interaction, thermal, mechanical, and invitro degradation of PLLA/CMS/β-TCP composites has been investigated under various electron-beam irradiation doses in the present work.

In tissue engineering, control of the degradation rate is one of the critical issues for synchronization of degradation kinetics to the rate of natural tissue formation. Control of the degradation rate is a challenging task. The novelty of this work is that electron-beam-induced PLLA/CMS/β-TCP composite nanofibers have been used to enhance the in vitro degradation of the PLLA matrix as an eco-friendly green method. The electron-beam irradiation doses and degradation rate-dependent effects on composite nanofibers have been examined.

## 2. Materials and Methods

Poly(l-lactide acid) with the inherent viscosity of 2.32 dl/g was obtained from BioInvigor (Taipei, Taiwan). β-TCP nanopowder was purchased from Berkeley Advanced Biomaterials Inc., USA, with an average particle size of approximately 250 nm. Carboxy-methyl starch was prepared from local sago starch. 10 g of sago starch was stirred in 300 mL isopropanol (Merck, GmbH, Darmstadt, Germany) with an addition to 30 wt.% NaOH in a reactor flask equipped with a reflux condenser and burette. In this work, the carboxymethylation process of sago starch was prepared as described by Bohari et al. [35]. Poly(l-lactide acid) solutions with 7 wt% mol concentration were prepared by dissolving the granule PLLA in dichloromethane by stirring the mixture for 24 h using a magnetic stirrer. The CMS solution was prepared at 10 wt% concentration and mix with PLLA in 5 % *v*/*v* of CMS content in PLLA. 0.25% of β-TCP powders were mixed in PLLA/CMS solution in the presence of sodium dodecyl sulfate (0.2 wt%). The samples were ultrasonic and stirred for 72 h and rotary-mixed to homogenize the mixture.

The PLLA/CMS/ β-TCP polymer solution was placed into a 1 mL syringe with a 0.6 mm diameter blunt needle tip. The distance between the needle and the collector was set as 12 cm and connected to a high voltage supplied at 10 kV (Gamma High Voltage Research Inc., Ormond, ES40P, 20 W, Ormond Beach, FL, USA). The syringe pump (New Era Pump System Inc. NE 1000, Toledo St, Farmingdale, NY, USA) was placed vertically with a flow rate of 0.006 mL/min, and the nanofiber mat was collected on aluminum foil.

### 2.1. Characterizations

#### 2.1.1. Surface Morphology

The surface of the fibers was characterized using a scanning electron microscope (SEM, Quanta 400, FEI, Dawson Creek Drive Hillsboro, Oregon, USA). The images were collected at ×2000 and ×10, 000 magnification at 10 kV. The fiber diameter was analyzed and calculated by using ImageJ (LOCI, University of Wisconsin, Madison, WI, USA) and Stack Graphic 4.0 software (Statgraphics Technologies, Inc.,The Plains, VA, USA).

#### 2.1.2. Chemical Interactions

Chemical interaction analysis was performed by using Fourier transform infrared (FTIR) spectroscopy (Perkin Elmer, Waltham, Massachusetts, USA). The FTIR spectrum was measured in the spectral range of 400 to 4000 cm^–1^, which was performed at 16 scans per sample.

#### 2.1.3. Thermal Behavior

Changes in thermal properties were investigated by using a differential scanning calorimeter (DSC, TA Instrument, Q20, New Castle, DE, USA). About 2.35 mg samples were heated from 20 °C to 250 °C at a heating rate of 10 °C /min. Nitrogen gas was purged to the sample approximately 35 mL min^–1^ to avoid oxidation during heating.

#### 2.1.4. X-ray Diffractions (XRD)

The crystalline and amorphous regions of samples were investigated using an XRD instrument (Bruker, AXS D8, Bremen, Germany). Samples were scanned from 5° to 60° of 2θ angle with a step size of 0.02°/sec. The X-ray source was from the CuKα with 1.5140 of the wavelength. Xpert High Score Plus software (Malvern Panalytical Ltd, Enigma Business Park, Grovewood Road, Malvern, UK) was used to analysis of the XRD.

#### 2.1.5. Wettability

Wettability of the nanofiber surfaces was determined by water-contact angle (WCA, One Attention Theta, TL100, Biolin Scientific, Espoo, Finland.). A 2 μL of distilled water was dropped on the surface of the nanofibers. 130 WCA data points were collected in 12 s.

#### 2.1.6. Mechanical Properties

The composite PLLA/CMS/β-TCP nanofibers were punched into a rectangular shape (5 × 15 mm^2^) with 10 mm of the gauge length for the tensile test. The samples were removed from the aluminum foil using a paper frame with double-sided tape attached to it. The frame provided additional support to the sample for handling during the testing process. Tensile testing was conducted using a 20 N load cell (Model UUK 5, Chungcheongbuk-do, Korea) equipped with a micro-stepper motor system (Ezi Step, Fastec, Bucheon, Republic of Korea) and an OMRON RXRX25 data logger to record the load. The elongation was determined by 1.0 mW Omron laser detector with detection limit of 2.5 ms/600 nm. The tensile test was conducted at 0.5 mm min^–1^ of the crosshead velocity. The tensile strength was taken as the maximum stress of the stress–strain curve.

#### 2.1.7. Irradiation

The samples were punched into a round disc of 12 mm diameter and placed in a sterile 12-well plate for in vitro degradation assessment. Composite nanofibers were irradiated into 3 different doses of 5, 30, and 100 kGy (EPS 3000, Nissin High Voltage, Chiyoda-ku, Tokyo, Japan) with 5 kGy per pass to reduce the heat generated from the electron beam. Voltage and current were set at 2 MeV and 1 mA, respectively. The distance between energy source and samples were set at 2 cm to optimize the electron alignment when hitting the samples.

#### 2.1.8. In Vitro Degradation

After irradiation, samples were immersed in a PBS medium for degradation study. The medium was refreshed every three days to provide fresh ions to the samples. Samples were incubated in the oven at 36.7 °C for 7, 14, 30, and 60 days. After 7, 14, 30, and 60 days, samples were removed and rinsed with deionized water before drying for 96 h at 45 °C. The weight of the samples was taken before and after immersion to obtain the percentage of mass loss after incubation.

## 3. Results

**Figure 1:**
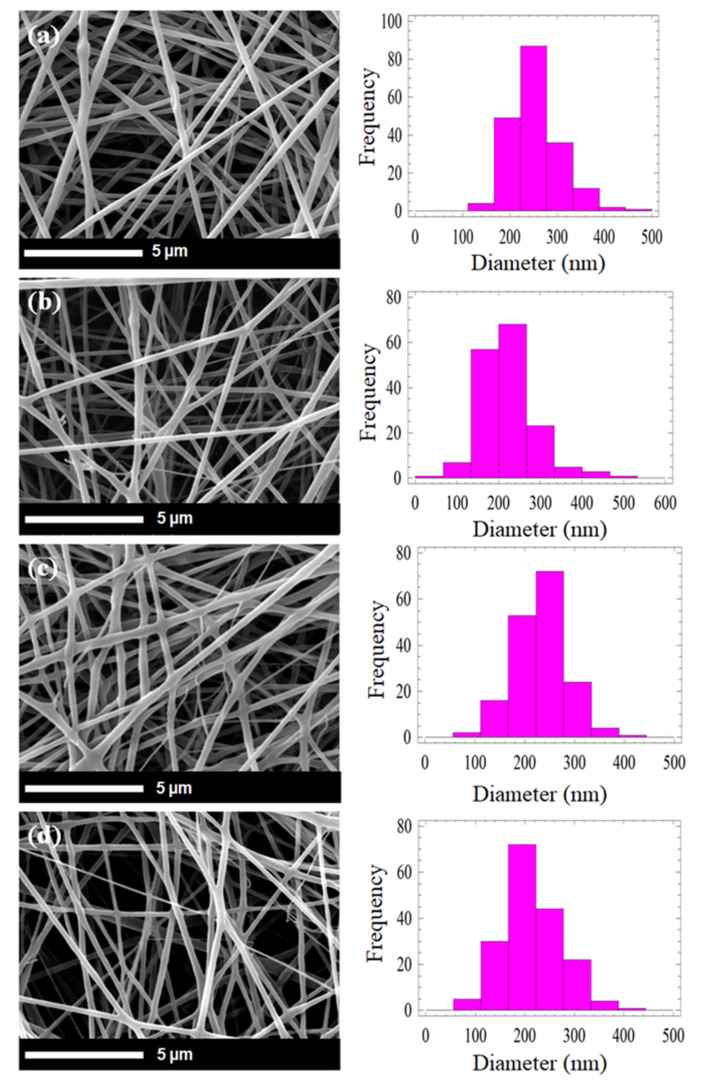
SEM micrographs of the morphology and fiber diameter distribution of PLLA/CMS/β-TCP irradiated at different irradiation doses (**a**) non-irradiated; (**b**) 5, (**c**) 30 and (**d**) 100 kGy.

**Figure 2:**
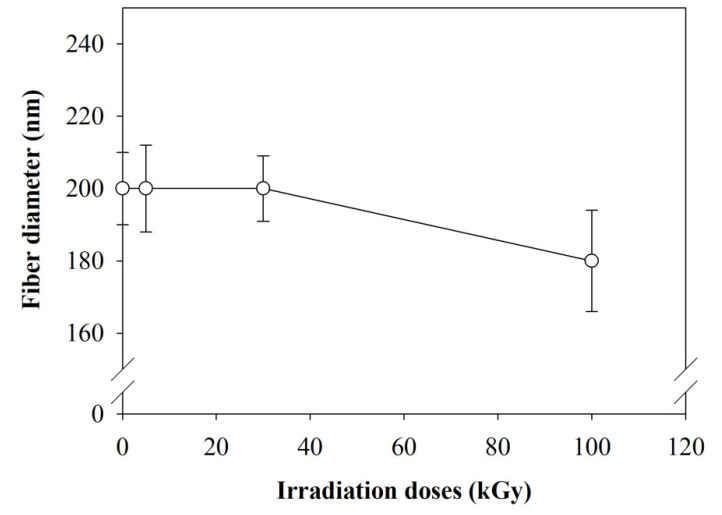
An average of fiber diameter with increase of irradiation dose.

Figure 1 shows the SEM images and fiber diameter distribution at different doses. Generally, there is no significant change in physical morphology at the lower irradiation doses. SEM images indicate no phase disintegration has been observed between the compositions of the composite nanofibers at both lower and higher doses of irradiation. Diameter distribution shows only a slight reduction of fiber diameter from 200 to 185 nm (Figure 2) at 100 kGy. Zhang et al. [32] reported a similar pattern of PLLA and PDLA. The fiber diameter reduced from 700 to 675 nm when the irradiation doses increase to 100 kGy. They also observed there was no crosslinking between PLLA and PDLA after irradiation. The possible reason for reduction in fiber diameter is due to the heat induced by the electron beam during bombarding [36]. The heat induced during irradiation leads to polymer shrinkages. The degradation of the polymer chains at higher doses may also contribute to slight reduction in average diameter of composite nanofibers [37]. As a result, the fiber diameter slightly reduces at high irradiation (100 kGy). By contrast, Lee at al. [29] suggested there was no influence on nanofiber diameter after PLGA was irradiated by an electron beam up to 300 kGy. However, Lee has not extended the discussion on insignificant influence of the high-dose electron beam to the PLGA nanofiber diameter.

**Figure 3:**
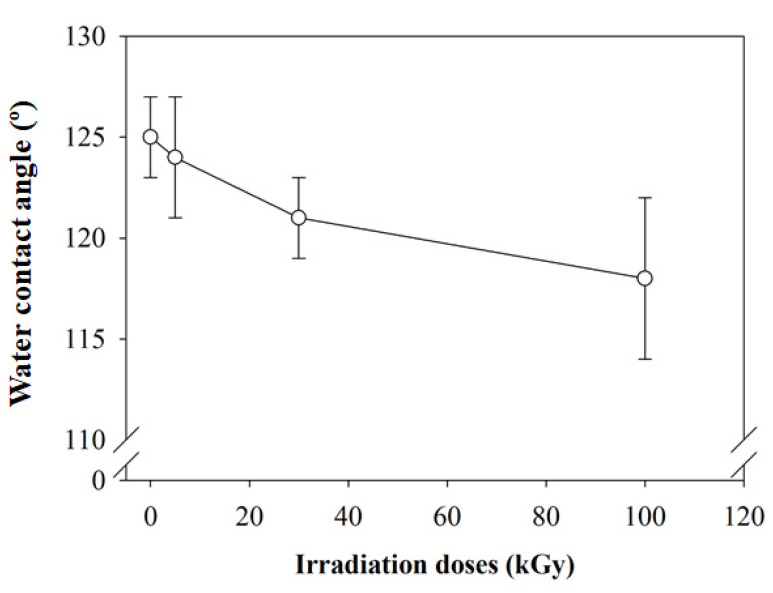
Changes in WCA with increase of irradiation doses.

The surface hydrophilic and hydrophobic behaviors of composite nanofibers were analyzed by water-contact angle measurement (WCA). The change in irradiation doses exhibited a small change in WCA. WCA reduced about 6 % from 125° to 118° after a 100 kGy irradiation dose (Figure 3). The possibility of slight reduce in WCA to 118° at 100 kGy was due to the formation of a hydrophilic group (OH) after degradation of the polymer chains at higher doses of irradiation. Even though CMS consists of a large amount of the hydroxyl group in the molecular structure, the 5% CMS composition in the composite may not contribute to enhance surface hydrophilic of the composite, as can be observed in the non-irradiated samples. Increased CMS to 20% was found to improve the surface hydrophilicity of the composite but destroyed the mechanical integrity of the scaffold, as shown our previous study [4]. The other factors, such as the surface texture and porosity of the nanofibers that might compete with the materials used, thus inhibit further reduction the WCA. The nanoscale surface texture encourages the water droplets to remain stable on the surface. However, the nature of the nanofibers with high porosity, which trapped the air inside, would inhibit the water from penetrating inside the nanofiber structure. By irradiating the composite nanofibers, surface wettability would be improved.

**Figure 4:**
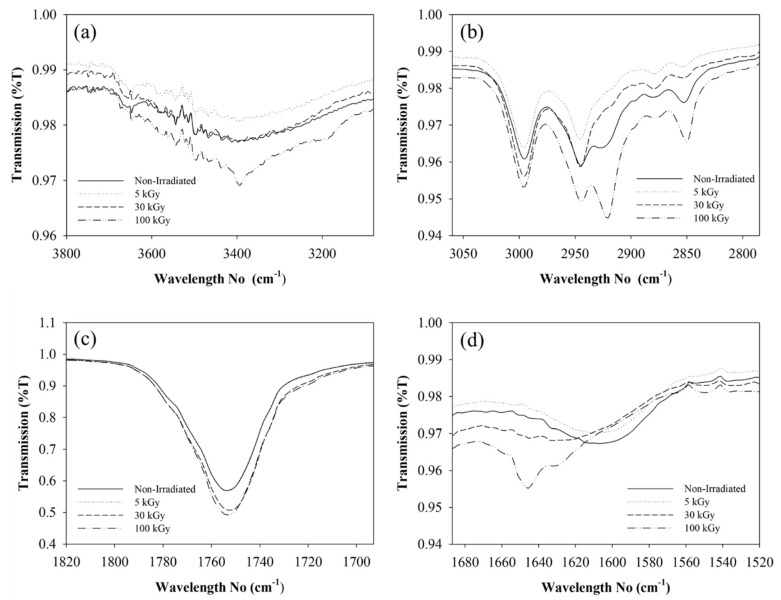
FTIR spectrum shows the chemical interaction in polymeric phase under different doses of electron-beam irradiation of (**a**) O–H bond, (**b**) C–H_3_, (**c**) C=O and (**d**) COO^–^.

Figure 4 shows the FTIR spectrum of the PLLA/CMSβ-TCP composite nanofibers at different irradiation doses. A change in intensity and shift in the absorption band was observed at 100 kGy. The absorption band intensity at 3600–3000 cm^–1^ has increased, indicating the amount of OH group formed from the degradation of the polymer chain in the CMS molecular structure (Figure 4a). CMS has an intense hydroxyl group bond in the backbone chain in the amylose and amylopectin group. There is no significant change in the hydroxyl absorption band at lower irradiation doses. The intensity of the absorption band of 2945 cm^–1^, which belongs to CH_3_ asymmetric stretching in PLLA, decreased at 5 and 50 kGy and increased at 100 kGy (Figure 4b). A similar pattern was observed in the CH bond at the 2850 cm^–1^ absorption band in PLLA. A decrease in the absorption band is suggested due to the interaction with the free radical that existed from the degradation of the polymer chain that attacks the bond. The increase in the absorption band of CH_3_ and CH at 100 kGy could be due to the degradation in the bonding of C–CH_3_ and C–CH in backbone PLLA structure. The increase in the ester bond (C=O) was also observed at 100 kGy at 1735 cm^–1^ of the absorption band (Figure 4c). The shifting of the COO–absorption band from 1607 to 1645 cm^–1^ in CMS indicates that the functional group interacted with the free radical at higher doses of irradiation (Figure 4d). Nagasawa et al. [38] observed crosslinking at 5 kGy in the CMS molecular structure, but degradation occurred between 20 to 40 kGy irradiation doses.

**Figure 5:**
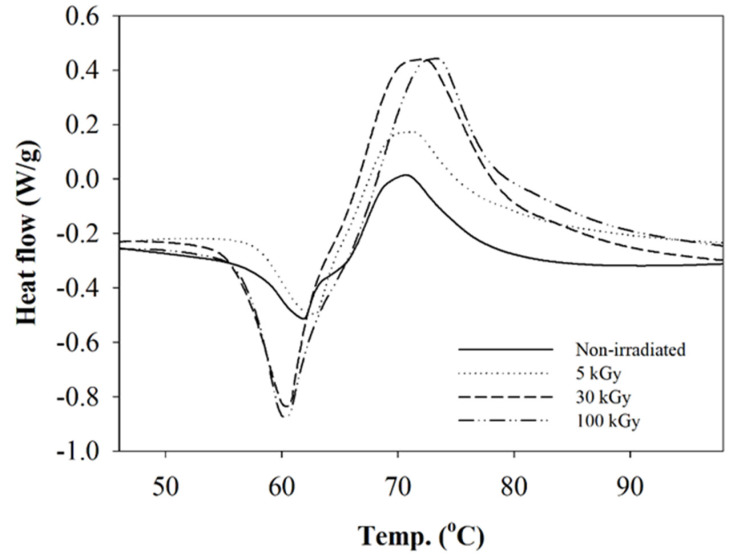
DSC thermogram exhibits the T_g_ and T_cc_ at different irradiation doses.

**Figure 6:**
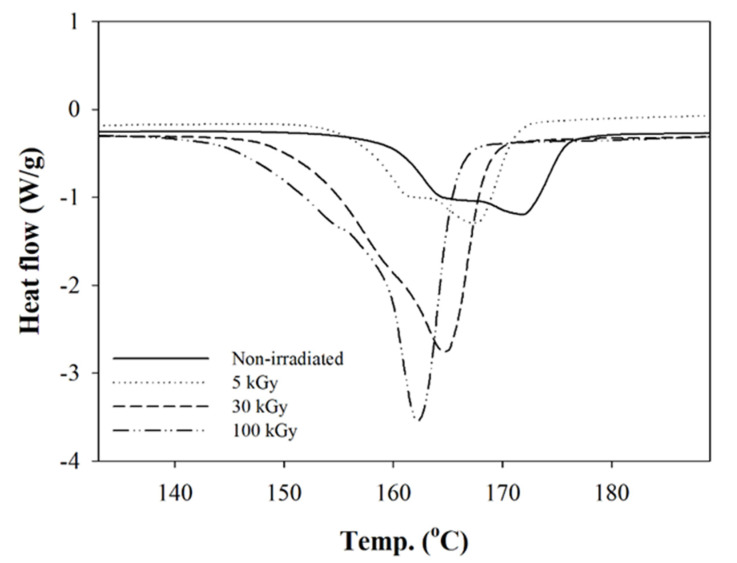
DSC thermogram exhibiting the Tm at different irradiation doses.

The thermal behavior of the composite nanofibers was investigated by DSC analysis. The glass transition temperature (T_g_), melting temperature (T_m_), and cold crystallization temperature (T_cc_) was determined by the DSC thermogram. The thermogram exhibited two endothermic peaks and one exothermic peak (Figure 5). The cold crystallization was shown by the presence of the endothermic peaks in the thermogram. The present of the T_cc_ peak indicated incomplete crystallization during the electrospinning process due to the fast drying and solidification of the polymer jet to form the nanofibers. The T_g_ reduced from 57.8° to 55.8° as irradiation doses increased from 5 to 100 kGy, but there was no significant change in T_g_ at 5 kGy (Figure 5). This indicates that no crosslinking occurred at this irradiation dose. Chain degradation is the dominant effect on composite nanofibers. A sharp peak of T_g_ at 30 and 100 kGy compared to 5 kGy was due to higher endothermic rate reflected from the chain degradation in the amorphous region. The exothermic peak of cold crystallization was observed in a sharp peak related to the higher rate of crystal rearrangement of the shorter chain from the degradation event. The arrangement of the imperfect crystal structure to a more perfect structure was also shown. As degradation takes place in the polymer molecular chain, the T_m_ reduces from 171.8° to 162.2° for non-irradiated and 100 kGy, respectively (Figure 6). The existing bi-modal peak at T_m_ occurs due to the presence of two types of crystal structure in a matrix composite (PLLA) during the electrospinning process, which were α and β, with a different melting temperature [39].

In summary, beam irradiation induced the degradation of composite nanofibers PLLA/CMS/β-TCP. The molecular chain of the polymer broke the shorter chain due to absorption of energy from the ionizing radiation by the atom and overcame the binding energy. This phenomenon occurred due to the elimination of excessive energy by breaking the bond at the exciting stage, which led to the formation of the alkyl radical from the matrix component (PLLA) at both amorphous and crystalline regions. At higher doses, degradation may occur by hydrogen breakage due to the higher oxygen permeability into the amorphous region [40]. Alkyl free radical interacts with oxygen to form peroxyl free radical and continues to cause the chain scission at the amorphous-crystalline interface [30].

**Figure 7:**
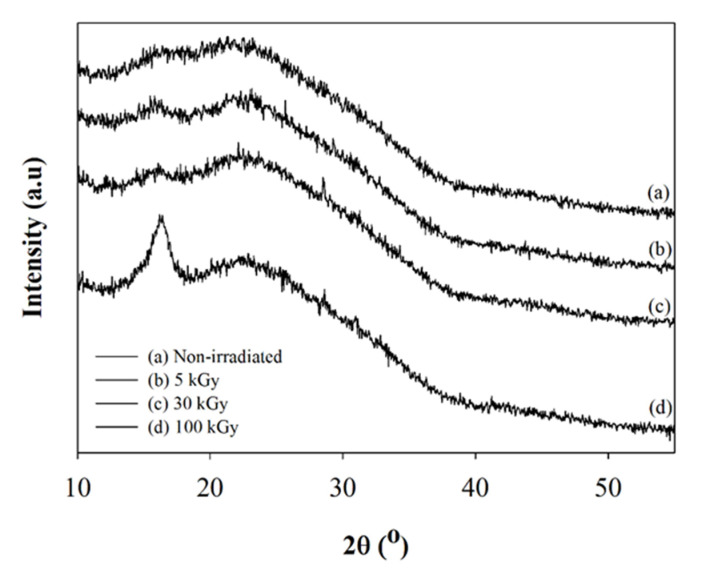
XRD diffractogram for PLLA/CMS/β-TCP composite nanofibers at different irradiation doses measured by DSC.

Figure 7 depicts the XRD diffractogram at different irradiation doses. There is no significant transformation observed at 5 and 30 kGy. The crystallization peak of the PLLA matrix presented at 2θ=16.2° due to rearrangement of the shorter chain to a crystalline form. The possible reason was due to the heat induced from the electron beam (EBIH) when the electron hit the surface of the composite nanofiber. While the sample was exposed to electron beam, elastic and inelastic interaction occurred. The energy generated during the inelastic interaction transformed into heat that caused the temperature to increase the composite nanofiber [41]. Although the study was carried out at the rate of 5 kGy per pass to reduce the heat effect, heat was not eliminated successfully at the 100 kGy dose. According to Cho et al. [42], the formation of α, α’, and β crystallite structure was formed in PLLA after exposure to heat and crystallite structure of α’, and β was not stable. A similar observation has been reported by Ryuji et al. [43] with PLLA after undergoing heat treatment. Kim et al. [40] observed the broadened peak of the XRD diffractogram after irradiation doses increased in PAN nanofibers. In this study, the rearrangement of the shorter chains to a crystallite structure was more rapid due to the increase of the heat and degradation of the polymer chain, which occurred concurrently. 

**Figure 8:**
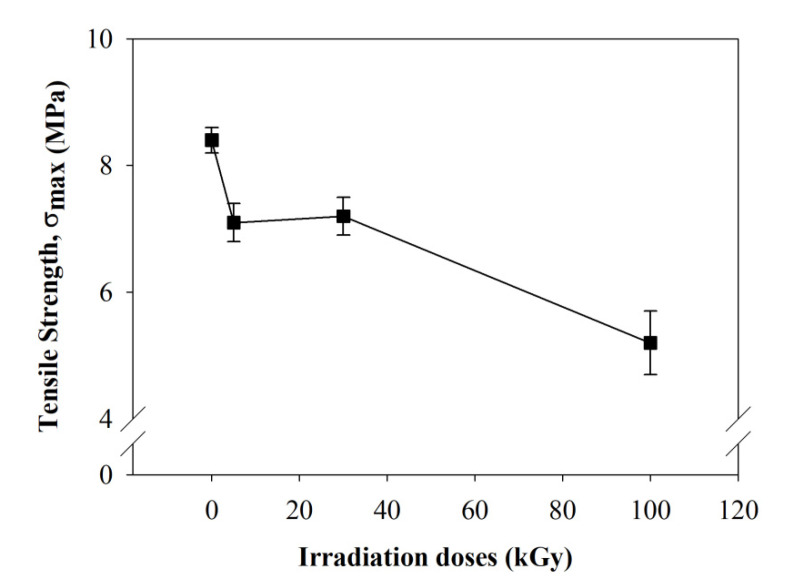
The effect of electron-beam irradiation to the tensile properties of PLLA/CMS/β-TCP nanofibers.

**Figure 9:**
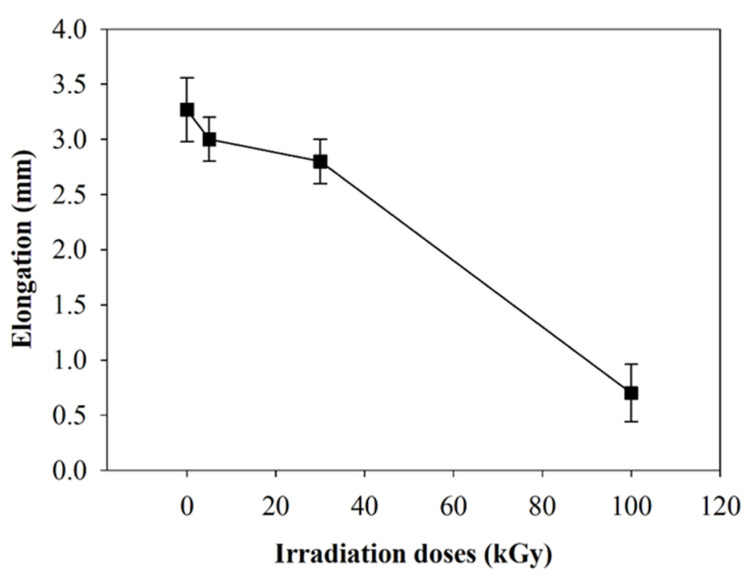
The effect of electron-beam irradiation to the elongation of PLLA/CMS/β-TCP nanofibers.

**Figure 10:**
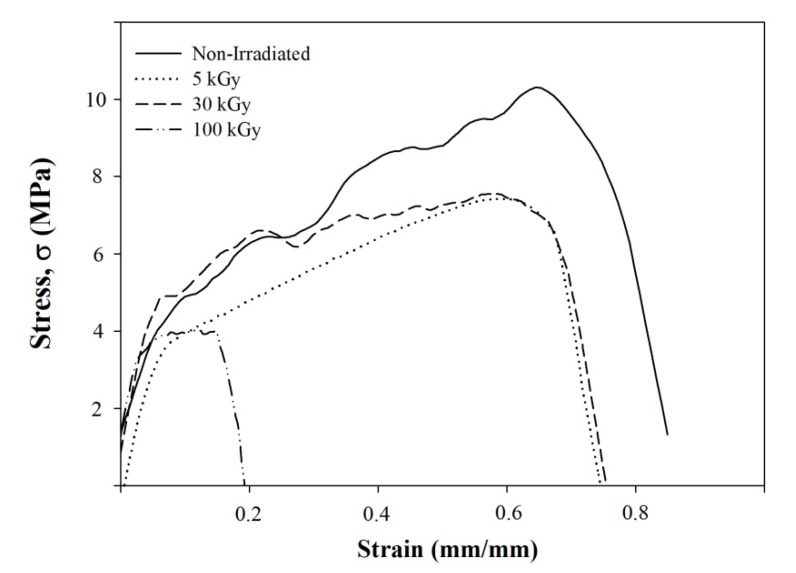
The effect of electron-beam irradiation to the breaking behavior of PLLA/CMS/β-TCP nanofibers.

Tensile strength was found to decrease with the increase of irradiation doses. Tensile strength reduced from 8.43 MPa for non-irradiated to 3.21 MPa after irradiation at 100 kGy (Figure 8). This could be related to the breaking of the polymer chain of irradiated composite nanofibers. A similar observation was reported by Jeun et al. [31], when composite nanofibers were irradiated in the range of 50 to 150 kGy. There was a rapid reduction of tensile strength observed after 100 kGy irradiation doses. Lee et al. [29] found that tensile strength reduced from 10 to 5.6 MPa after PLLA was irradiated in the range of 50 to 150 kGy. Lee suggested the reduction in tensile strength was caused by the degradation of the polymer chain based on the reduction of PLLA molecular weight. The elongation of PLLA/CMS/β-TCP composite nanofibers was also found to decrease from 3.2 to 0.6 mm for non-irradiated and 100 kGy doses (Figure 9). That could be related to the degradation of the polymer chain. The plastic behavior of nanofibers decreased as brittle failure took place at higher-irradiation doses (Figure 10). The rearrangement of the polymer chain to the crystallite structure from the broken chain contributed to brittle behavior without any plastic region during the tensile test. Based on the XRD diffractogram, the orientation of the shorter chain might occur at the diffraction peak of 16.2° presented in 100 kGy in the PLLA matrix. However, the form of crystallization did not contribute to the strengthening of the structure due to the polymer chain in the amorphous region being related to degradation via chain scission.

**Figure 11:**
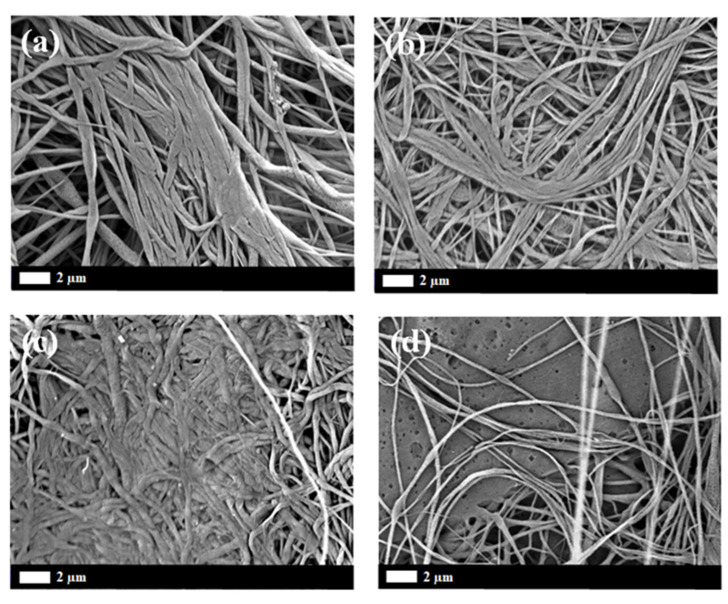
SEM micrograph of the PLLA/CMS/β-TCP morphology after 30 days immersion at different irradiation doses: (**a**) non-irradiated; (**b**) 5 kGy; (**c**) 30 kGy; and (**d**) 100 kGy.

**Figure 12:**
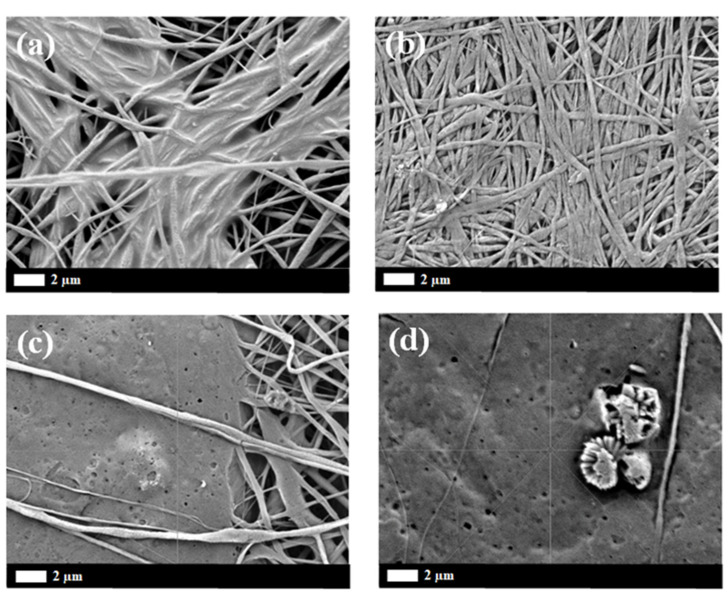
SEM micrograph of the PLLA/CMS/β-TCP morphology after 60 days immersion at different irradiation doses: (**a**) non-irradiated; (**b**) 5 kGy; (**c**) 30 kGy; and (**d**) 100 kGy.

**Figure 13:**
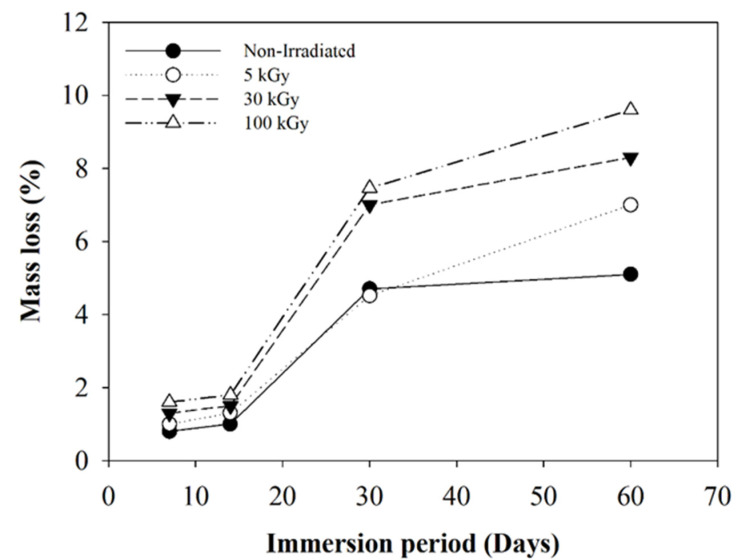
Mass loss of the PLLA/CMS/β-TCP exposed under different irradiation doses of electron beam after 60 days immersion in PBS medium.

Investigation of in vitro degradation was carried out by immersing the composite nanofibers into PBS solution for 7, 14, 30, and 60 days. Changes in morphology and the mass loss indicated the degradation of composite nanofibers. Figure 11 and Figure 12 show the SEM micrograh of the composite nanofiber morphology after 30 and 60 days immersion in SBF (Simulated Body Fluid). The morphology of the composite nanofibers before PBS solution immersion is shown in Figure 1. Morphology exhibited a swelling of the individual fibers after 30 days immersion for non-irradiated and 5 kGy-irradiated nanofibers. The obvious swelling and contraction of each individual fiber can be observed at 30 kGy (Figure 11b). The breaking of the polymer chains enhanced the medium molecule to penetrate and attack the molecular chain. The “melt” fibers can be observed at 100 kGy as breaking the molecular chain at the amorphous region to mobilize the chain as a plastic behavior (Figure 11d). It can be observed as no breaking fibers at 100 kGy. After 60 days immersion, the melt and the swelling fibers covered the pores for non-irradiated and 5 kGy-irradiated fibers. There was no observed pore at 5 kGy (Figure 12b). Most of the fibers were melted at 30 kGy and completely melted at 100 kGy. At higher-irradiation doses, more of the chain broke into small fragments and enhanced the penetration of the medium molecule to attack the bond. The small fragments decomposed into the oligomer form that dissolved into medium, as can be observed in the mass loss. The most obvious mass loss was observed at 30 and 60 days. The percentage of mass loss is between 1–9% for immersion until 60 days (Figure 13). More chain break in shorter fragmentation would speed up the process to form an oligomer and dissolve into medium. This can be observed at 100 kGy irradiation doses with the highest percentage of mass loss. According to Zulkifli et al. [44], hydrolysis occurs at the segmentation of the molecular chain to form the oligomer during in vitro degradation that could be dissolved in a medium. Here, we prove that electron-beam irradiation helps to decompose composite PLLA/CMS/β-TCP nanofibers, which takes the process a step further towards using nanofiber mats in biomedical applications.

## 4. Conclusions

Electron-beam-induced composite PLLA/CMS/β-TCP nanofiber mats have been prepared in order to analyze the beam effect at various doses (5, 30, and 100 kGy) on the physical and chemical morphology and degradation behavior of materials. SEM and WCA were used to evaluate surface morphology while FTIR, DSC, and XRD were used for the chemical morphology of the composite nanofibers. Furthermore, tensile and in vitro degradation tests were run to evaluate the degradation of samples under various beam intensities. The results can be concluded as follows:

- the fiber diameter of composite nanofibers changed slightly after beam irradiation.
- WCA dropped from 125 to 118° due to the formation of hydroxyl groups after the highest dose of beam irradiation.
- FTIR, DSC, and XRD results indicated that there are chemical changes to the structure of composite nanofibers after the highest dose of beam irradiation.
- A tensile test was run to observe the mechanical strength before and after beam irradiation. Apparently, the material decreased its mechanical properties by 2.6 times due to the breaking of the polymer chains after the highest beam-irradiation dose. Moreover, rapid reduction in the elongation showed that polymer degradation took place after beam irradiation.
- SEM images after electron-beam irradiation showed that degradation took place after 30 and 60 days in vitro test.
- Electron-beam irradiation enhanced the in vitro degradation of PLLA matrix since the PLLA component has a longer degradation period. However, higher-irradiation doses retarded the mechanical integrity of the composite.

This method to prepare degradable nanofibers is helping to develop and design new biomaterials. Electron-beam-irradiated composite PLLA/CMS/β-TCP nanofiber mats showed rapid biodegradation demonstrating their potential for tissue-engineering applications, specifically bone tissue.
